# Preoperative chemoradiotherapy with the TEGAFIRI regimen achieves significant local control in locally advanced rectal cancer

**DOI:** 10.1007/s00384-025-04867-0

**Published:** 2025-03-26

**Authors:** Shigenobu Emoto, Kazushige Kawai, Koji Oba, Hiroaki Nozawa, Kazuhito Sasaki, Koji Murono, Yuichiro Yokoyama, Shinya Abe, Kensuke Kaneko, Yuzo Nagai, Takahide Shinagawa, Yuichi Tachikawa, Satoshi Okada, Soichiro Ishihara

**Affiliations:** 1 Department of Surgical Oncology, The University of Tokyo, 7-3-1, Hongo, Bunkyo-Ku, Tokyo, 113-8655 Japan; 2 Department of Surgery, Tokyo Metropolitan Cancer and Infectious Disease Center, Komagome Hospital, Tokyo, Japan; 3 Department of Biostatistics, The University of Tokyo, Tokyo, Japan

**Keywords:** TEGAFIRI regimen, Preoperative chemoradiotherapy, Locally advanced rectal cancer

## Abstract

**Purpose:**

This study aims to evaluate both the short- and long-term outcomes of preoperative chemoradiotherapy (CRT) using the tegafur-uracil/calcium folinate/irinotecan (TEGAFIRI) regimen in patients with locally advanced rectal cancer (LARC). While total neoadjuvant therapy (TNT) is becoming more common, CRT may still be the optimal approach in certain cases to improve prognosis and reduce adverse events.

**Methods:**

This single-center, retrospective cohort study included patients with histologically confirmed nonmetastatic primary adenocarcinoma of the lower rectum treated with preoperative CRT using the TEGAFIRI regimen (TEGAFIRI group). The control group comprised patients treated with tegafur-uracil/calcium folinate (UFT group). The primary endpoint was the pathologic complete response (pCR) rate. Secondary endpoints included adverse events, overall survival (OS), disease-free survival (DFS), distant recurrence-free survival (DRFS), and local recurrence-free survival (LRFS). The background was adjusted using inverse probability weighting (IPW) calculated with the propensity score.

**Results:**

The TEGAFIRI group consisted of 79 patients, while the UFT group included 264. The standardized pCR rates through the IPW were as follows: TEGAFIRI group: 24.3%, UFT group: 8.8%, and the difference in pCR was 15.4% (*P* = 0.01). Adverse events of grade 3 or higher were observed in 15.2% vs. 8.7% (adjusted) (13.6% vs. 9.1% crude) in the TEGAFIRI group and the UFT group. The standardized LRFS was significantly higher in the TEGAFIRI group (HR = 0.39, (95% CI 0.16–0.98), *P* = 0.045). There were no significant differences in OS, DFS, or DRFS between groups.

**Conclusions:**

The TEGAFIRI regimen for preoperative CRT in LARC demonstrated a high pCR rate and reduced local recurrence, with manageable adverse events.

## Introduction

Treatment of locally advanced rectal cancer (LARC) traditionally involves preoperative chemoradiotherapy (CRT) followed by surgery, specifically total mesorectal excision (TME), which has been established as the standard of care [1, 2]. Although preoperative CRT has reduced the local recurrence (LR) rate to approximately 5%, the distant recurrence rate remains at approximately 30%, with no significant improvement in overall survival (OS).

Total neoadjuvant therapy (TNT) has garnered attention as a novel approach for the treatment of LARC. TNT involves the administration of adjuvant chemotherapy before surgery, either before or after CRT, to enhance patient compliance, reduce distant metastases, improve local control, and ultimately extend patient survival [3–5]. TNT is particularly recommended for cases requiring tumor shrinkage or sphincter-preserving surgery. Moreover, the concept of non-operative management is gaining traction for patients who respond well to preoperative treatment, delaying surgery, as demonstrated in the OPRA trial [6]. In this context, patients achieving clinical complete remission can be managed with a watch-and-wait approach; however, the increased incidence of adverse events is of concern. Additionally, prolonging the interval to surgery may exacerbate radiation-induced fibrosis, complicating accurate TME and potentially increasing LR [7]. Therefore, it is crucial to tailor treatment based on disease progression and patient performance status to avoid unnecessary TNT administration.

Various combination regimens have been explored in preoperative CRT to enhance local control. Oral fluoropyrimidine-based agents are the standard treatment for patients undergoing concurrent chemotherapy. Numerous trials have tested the addition of oxaliplatin but have reported inconsistent results. Although the CAO/ARO/AIO-04 trial demonstrated improved disease-free survival (DFS) possibly related to better treatment adherence [8], most other trials reported increased hematologic toxicity and other adverse events. Only two phase III trials involving irinotecan have been conducted; the ARISTOTLE trial did not demonstrate a significant increase in the pathologic complete response (pCR) rates with irinotecan treatment (17% vs. 20%, *P* = 0.45) [9]; however, a Chinese study comparing TNT with capecitabine plus radiotherapy and CAPOX consolidation chemotherapy versus CAPIRI showed a significant increase in the pCR rates (15% vs. 30%, *P* < 0.001) in addition to increased hematological toxicity (6% vs. 38%, *P* < 0.001) [10]. Developing regimens that can increase pCR rates while minimizing adverse events remains critical.

We previously conducted a phase I/II clinical trial demonstrating that the TEGAFIRI regimen achieved high pCR rates with low toxicity [11]. This biweekly irinotecan administration approach is novel, aiming to enhance radiosensitization and reduce adverse events. Our previous report presented data from a single-arm study involving a small number of patients. Therefore, it is necessary to clarify the extent of the benefits provided by TEGAFIRI compared with existing therapies with a larger number of patients.

This study aimed to validate the efficacy and safety of preoperative CRT with the TEGAFIRI regimen and compare it with the conventional tegafur-uracil (UFT)/calcium folinate (LV) regimen. We evaluated the adverse events, surgical outcomes, pathological response, and survival rates.

## Method

### Patients and study design

Beginning in October 2018, we prospectively enrolled patients who underwent preoperative CRT followed by TME for LARC in our department. The CRT regimen of choice was TEGAFIRI, and we analyzed the cases in which primary tumor resection was completed by September 2023. The treatment protocol was based on that used in our previous phase I/II clinical trial [11]. Specifically, patients aged 20–80 years with histologically confirmed nonmetastatic primary adenocarcinoma (well or moderately differentiated) of the lower rectum (cT3–cT4, any N) were eligible. Patients with M1 disease were included only if the distant metastases were within the radiation field and resectable, specifically inguinal lymph node metastases or, in some cases, para-aortic lymph node metastases. Additional eligibility criteria included an Eastern Cooperative Oncology Group (ECOG) performance status of 0 or 1; normal liver, renal, heart, and bone marrow function; and written informed consent. The *UGT1A1* genotype was tested before treatment initiation; those with wild-type (–/–) or single heterozygous (–/*6 or –/*28) status were included. The exclusion criteria were double heterozygous (*6/*28) or homozygous (*6/*6 or *28/*28) *UGT1A1* status, previous chemotherapy for rectal cancer, a history of malignant disease within 5 years, and severe diarrhea or uncontrolled infection.

In total, 79 consecutive patients received preoperative CRT with the TEGAFIRI regimen and were enrolled. Clinical and pathological data were extracted from the medical records. The control group included 264 patients with LARC who underwent preoperative CRT with the conventional UFT/LV regimen, followed by TME, between February 2005 and September 2018.

### Treatment protocol

Radiotherapy (RT) commenced on the first day of chemotherapy, administered five times weekly with a daily fraction of 1.8 Gy. The entire pelvis was treated using a 3- or 4-field technique with a total dose of 50.4 Gy, using a 10-MV X-ray accelerator in the supine position. The clinical target volumes included the entire pelvic cavity, anal canal, primary tumor, mesorectal and presacral lymph nodes, lymph nodes along the internal iliac artery, lumbar nodes up to the lower border of the fifth lumbar vertebra, and obturator lymph nodes. The superior border is the bifurcation of the internal and external iliac arteries. UFT (300 mg/m^2^/day) and LV (75 mg/body weight/day) were administered orally three times daily on days 1–5, 8–12, 15–19, 22–26, and 29–33. Irinotecan was administered intravenously at 80 mg/m^2^ on days 1, 15, 29, and 43. No dose-limiting protocol was applied. During CRT and the interval between RT and surgery, patients were examined every 1–2 weeks, and adverse events were graded according to the Common Terminology Criteria for Adverse Events from the National Cancer Institute (version 5.0). Appropriate medications were administered in cases of adverse events.

Radical surgery was performed 6–12 weeks after CRT completion. Total or tumor-specific mesorectal excision was performed, along with selective lateral lymph node dissection for suspected metastasis. Specifically, lateral lymph nodes (LLNs) with a long axis ≥ 8 mm on pre-CRT computed tomography scans were dissected regardless of post-CRT size [12, 13]. All resected specimens were subjected to histopathological analyses. Pathological TNM classification and staging were determined using the 8th edition of the American Joint Committee on Cancer guidelines [14]. Tumor regression grade (TRG) was assessed using the Japanese Classification of Colorectal Carcinomas, with complete regression (grade 3) defined as the absence of viable cancer cells. Regression exceeding two-thirds of the tumor volume was classified as grade 2. Tumors with regression in less than two-thirds were classified as grade 1 (grade 1a: < 1/3, grade 1b: ≥ 1/3 but < 2/3), and no regression was classified as grade 0. pCR was defined as complete disappearance of the cancer in the primary lesion and lymph nodes and no distant metastases [15]. The control group (UFT group) received the same treatment but without irinotecan. A 6-month adjuvant chemotherapy regimen with CAPOX was generally recommended. Based on the patient’s age, physical condition, and preferences, adjustments were considered, including shortening the duration to 3 months, switching to single-agent capecitabine or UFT/LV, or omitting adjuvant chemotherapy altogether.

### Endpoints

The primary endpoint was pCR. Secondary endpoints included adverse events, OS, DFS, distant recurrence-free survival (DRFS), and local recurrence-free survival (LRFS).

### Statistical analyses

Fisher’s exact test evaluated the relationships between clinicopathological features and treatment. Nonparametric comparisons were performed using the Wilcoxon test.

The propensity score (PS), defined as the probability of a patient receiving TEGAFIRI conditional on the observed confounders, was estimated using logistic regression with the following variables: age at the start of treatment, sex, clinical T stage (T4 or other), clinical mesorectal lymph node metastasis (positive or negative), clinical lateral lymph node metastasis (positive or negative), and the interval from CRT completion to surgery, which was log-transformed and denoted as ln_time. The inverse probability of treatment weight (IPW), calculated as 1/PS for patients who received TEGAFIRI and 1/(1 – PS) for patients who received conventional treatment (UFT group), was used to estimate the causal average treatment effect (ATE) in the overall population. The balance of confounders before and after the IPW analysis was assessed using weighted standardized differences between the TEGAFIRI and UFT groups. Differences in the means of confounders were considered negligible if they were below the threshold of 0.1 standard deviations [16]. The PS distribution was also graphically compared between groups.

For the primary analysis, the standardized pCR rates through IPW and their differences were compared between the TEGAFIRI and UFT groups. A 95% confidence interval (CI) was estimated using 10,000 bootstrap samples. For the secondary endpoints, weighted Kaplan–Meier curves were generated for the time-to-event outcomes, and weighted Cox regression analysis was used to estimate the hazard ratio between the TEGAFIRI and UFT groups. In addition, prognostic factors for all patients were analyzed using the Cox regression model. We tested the proportional hazards assumption using Schoenfeld residuals with the cox.zph function from the survival package in R and found no violations of the assumption. Statistical analyses were performed using CAUSALTRT in SAS 9.4 software (SAS Institute, Cary, NC, USA) and R version 4.4.1 (R Foundation for Statistical Computing, Vienna, Austria).

## Results

### Patient characteristics and IPW

**Fig. 1:**
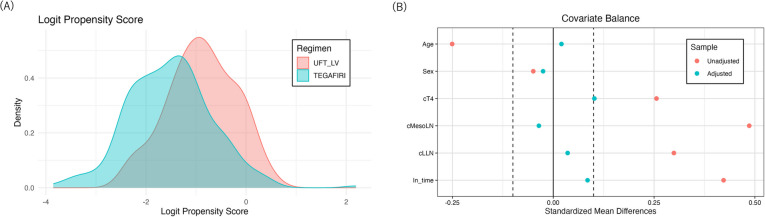
Graphical check of the propensity score and inverse probability weight. **A** Check of overlap of the propensity score. **B** Standardized mean difference of confounders before/after inverse probability weighting

Patient characteristics of the TEGAFIRI and UFT groups are shown in Table 1. Since there were only two patients in the TEGAFIRI group and five patients in the UFT group with cT2 or lower, the cT stage was stratified into cT1-3 and cT4. There was one patient with cT1cN + in each group, one patient with cT2cN + in the TEGAFIRI group, and four patients in the UFT group. The TEGAFIRI group had a significantly higher proportion of N-positive cases in both the mesenteric and lateral lymph nodes. Each group included three and four M1 cases, respectively, indicating metastases to the para-aortic or inguinal lymph nodes, which were included in the radiation field and considered resectable. The analysis utilized IPW based on PS to achieve covariate balance. The overlap in the distribution of the logit PS is shown in Fig. 1A. There was an overlap in the PS range between the TEGAFIRI and conventional UFT groups. We also checked the balance of confounders before and after the IPW analysis (Fig. 1B). Although the adjusted mean difference for cT4 slightly exceeded the 0.1 threshold (0.102), this imbalance was considered acceptable, as all other covariates achieved adequate balance (mean differences < 0.1). Therefore, the analysis was conducted with the assumption that the overall covariate balance was sufficiently achieved.

**Table 1:** Patients’ characteristics

Variable	UFT	TEGAFIRI	Weighted UFT	Weighted TEGAFIRI	*P* (crude)	*P* (weighted)
*N*	264	79	351	320		
Age, mean (SD)	63.2 (10.8)	60.4 (11.6)	62.8 (10.9)	63.0 (11.2)	0.050	0.89
Sex, female (%)	94 (35.6)	30 (38.0)	123 (35.0)	116 (36.1)	0.80	0.86
BMI, kg/m^2^, mean (SD)	22.8 (3.56)	24.1 (4.70)	22.8 (3.57)	23.8 (4.24)	0.006	0.08
cT4 (%)	24 (9.1)	14 (17.7)	40.1 (11.4)	47.7 (14.9)	0.052	0.45
cMesoLN metastasis (%)	113 (42.8)	53 (67.1)	173 (49.3)	152 (47.5)	< 0.001	0.80
cLLN metastasis (%)	48 (18.2)	24 (30.4)	71.5 (20.4)	70.0 (21.8)	0.029	0.79
Distance from AV, cm, mean (SD)	4.30 (2.51)	4.35 (2.51)	4.23 (2.49)	4.44 (2.49)	0.89	0.55
Ln_time, mean (SD)	4.02 (0.30)	4.15 (0.14)	4.09 (0.40)	4.12 (0.14)	< 0.001	0.63

*SD* standard deviation, *CI* confidence interval, *BMI* body mass index, *cT* clinical T, *cMesoLN* clinical mesorectal lymph nodes, *cLLN* clinical lateral lymph nodes, *AV* anal verge, *Ln_time* log-transformed interval between chemoradiation and surgery

### Tolerability

The completion rate of CRT without dose reduction or delay was 63% and 95.5% in the TEGAFIRI and conventional treatment groups, respectively (*P* < 0.0001). In the TEGAFIRI group, the relative dose intensities (RDIs) for irinotecan, UFT, and RT were as high as 0.895 ± 0.182, 0.977 ± 0.066, and 0.996 ± 0.025, respectively (mean ± SD). In the conventional treatment group, the RDIs for UFT and RT were 0.983 ± 0.10 and 0.995 ± 0.047, respectively, with no significant differences between groups.

### Adverse events

The main adverse events during preoperative CRT, adjusted for baseline differences using IPW, are summarized in Table 2. In the TEGAFIRI group, 15.2% of adjusted patients (13.6% of crude patients) experienced grade 3 or higher adverse events, including leukopenia (11.7%), neutropenia (7.2%), and diarrhea (3.3%). In the UFT group, 8.7% of adjusted patients (9.1% of crude patients) experienced grade 3 or higher adverse events.

**Table 2:** Incidence and risk difference of adverse events

Adverse event	Severity	Weighted UFT incidence, % (95% CI)	Weighted TEGAFIRI incidence, % (95% CI)	Risk difference, % (95% CI)	*P*
Leukopenia	All grade	37.6 (31.4–43.8)	50.9 (38.2–63.5)	13.3 (− 0.9–27.4)	0.057
	Grade 3 ≥	2.2 (0.4–3.9)	11.7 (2.5–20.9)	9.5 (1.0–19.3)	0.044
Neutropenia	All grade	29.3 (22.6–36.0)	36.6 (24.5–48.8)	7.3 (− 5.7–21.4)	0.29
	Grade 3 ≥	1.9 (0.2–3.6)	7.2 (0.3–14.2)	5.3 (− 1.0–13.5)	0.14
Anemia	All grade	77.4 (72.2–82.5)	92.9 (86.9–98.9)	15.6 (7.4–23.1)	< 0.001
	Grade 3 ≥	1.3 (0.0–2.5)	0.5 (0.0–1.6)	− 0.7 (− 2.4–0.9)	0.38
Thrombocytopenia	All grade	24.7 (19.4–30.1)	19.9 (9.9–29.8)	− 4.9 (− 15.8–7.0)	0.40
	Grade 3 ≥	0.0 (0.0–0.0)	0.0 (0.0–0.0)	NaN	NaN
AST elevation	All grade	12.6 (8.6–16.6)	30.5 (18.8–42.2)	17.9 (5.8–30.5)	0.005
	Grade 3 ≥	1.9 (0.2–3.6)	0.7 (− 0.7–2.0)	− 1.2 (− 3.4–1.0)	0.27
ALT elevation	All grade	15.4 (11.0–19.8)	39.5 (27.2–51.7)	24.1 (11.4–37.8)	< 0.001
	Grade 3 ≥	2.2 (0.4–3.9)	0.7 (− 0.7–2.2)	− 1.4 (− 3.7–0.9)	0.22
Nausea	All grade	12.8 (8.6–17.1)	58.5 (45.6–71.4)	45.7 (32.4–59.5)	< 0.001
	Grade 3 ≥	1.0 (0.0–2.2)	0.0 (0.0–0.0)	− 1.0 (− 2.4–0.0)	0.08
Diarrhea	All grade	47.1 (40.4–53.7)	87.8 (79.5–96.0)	40.7 (29.6–50.9)	< 0.001
	Grade 3 ≥	1.1 (0.0–2.3)	3.3 (0.3–6.3)	2.3 (− 0.7–5.9)	0.18
Anal pain	All grade	44.8 (38.3–51.3)	71.1 (60.1–82.0)	26.3 (13.3–38.4)	< 0.001
	Grade 3 ≥	1.5 (0.0–3.0)	0.0 (0.0–0.0)	− 1.5 (− 3.2 to − 0.3)	0.048
Total	All grade	96.2 (94.0–98.4)	100 (100–100)	3.8 (1.7–6.1)	< 0.001
	Grade 3 ≥	8.7 (5.3–12.1)	15.2 (5.7–24.7)	6.5 (− 2.8–16.9)	0.20

*CI* confidence interval, *NaN* not a number, *AST* aspartate aminotransferase, *ALT* alanine aminotransferase

### Surgical outcome

Surgical outcomes are shown in Table 3. The rate of minimally invasive surgery was significantly higher in the TEGAFIRI group, likely due to historical factors. The most common morbidity was pelvic dead space infection, followed by small bowel obstruction. Anastomotic leakage did not occur in any patients in the UFT group, whereas it was observed in 2 patients (2.5%) in the TEGAFIRI group. Postoperative 30-day mortality was zero in both groups.

**Table 3:** Surgical outcomes

Outcome	Weighted UFT, % or median (95% CI)	Weighted TEGAFIRI, % or median (95% CI)	Risk difference, % or median (95% CI)	*P*
MIS	65.8 (59.1–72.4)	92.8 (86.5–99.2)	27.1 (17.8–35.9)	< 0.001
Anal preservation	71.1 (64.3–77.8)	71.2 (59.8–82.6)	0.1 (− 13.0–13.0)	0.98
Lateral pelvic node dissection	18.6 (13.5–23.7)	18.6 (11.0–26.6)	0.0 (− 9.0–10.0)	1.00
Operative time (min, median)	349 (336–370)	376 (349–474)	27 (− 12.4–120)	0.40
Blood loss (g, median)	200 (150–280)	87.3 (50–126)	− 113 (− 190 to − 50)	< 0.001
Morbidity (CD ≥ 3)	7.2 (3.9–10.4)	10.6 (3.2–18.0)	3.4 (− 4.1–12.2)	0.41
Post op. hospital stay (days, median)	19 (18–20)	17 (15–19)	− 2 (− 4–0)	0.06
Adjuvant chemotherapy	40.5 (33.7–47.3)	58.3 (45.9–70.6)	17.8 (3.4–31.7)	0.01

*CI* confidence interval, *MIS* minimally invasive surgery, *CRT* chemoradiotherapy, *LAR* low anterior resection, *ISR* intersphincteric resection, *APR* abdominoperineal resection, *CD* Clavien–Dindo, *SBO* small bowel obstruction, *SOO* stoma outlet obstruction, *SSI* surgical site infection

### Pathological outcome

The histological response grades 0/1a/1b/2/3 were observed in the following number of patients: 0, 6, 15, 42, and 16 in the TEGAFIRI group, and 1, 51, 78, 109, and 25 in the UFT group, respectively. One patient in the UFT group had residual cancer cells in the inguinal lymph nodes. The crude pCR rates were 19.8% for the TEGAFIRI group and 9.1% (24 cases) for the UFT group, with a significantly higher rate in the TEGAFIRI group. The comparison adjusted by inverse probability weighting (IPW) is shown in Table 4. The postoperative pathological staging for the patients in both treatment groups is as follows: In the UFT group, the distribution of pathological stages was as follows: Stage 0: 23 (8.7%), Stage 1: 74 (28.0%), Stage 2: 83 (31.4%), Stage 3: 69 (26.1%), and Stage 4: 15 (5.7%). In the TEGAFIRI group, the distribution was as follows: Stage 0: 16 (20.3%), Stage 1: 25 (31.7%), Stage 2: 16 (20.3%), Stage 3: 18 (22.8%), and Stage 4: 4 (5.1%).

**Table 4:** Pathological outcomes

Outcome	Weighted UFT, % (95% CI)	Weighted TEGAFIRI, % (95% CI)	Risk difference, % (95% CI)	*P*
Pathological MesoLN metastasis	27.7 (21.9–33.5)	18.9 (10.3–27.6)	− 8.8 (− 19.0 to − 2.0)	0.10
Pathological LLN metastasis	9.4 (5.5–13.4)	7.2 (2.3–12.1)	− 2.2 (− 8.3–4.3)	0.49
CRM positive or close	5.0 (0.0–10.1)	1.2 (0.0–3.0)	− 3.8 (− 10.9–1.1)	0.20
TRG ≥ 2	48.8 (42.1–55.3)	73.2 (61.7–84.8)	24.5 (10.9–37.7)	< 0.001
pCR rate	8.8 (5.4–12.3)	24.3 (12.9–35.7)	15.4 (4.3–27.8)	0.01

CI confidence interval, *TRG* tumor regression grade, *MesoLN* mesorectal lymph nodes, *LLN* lateral lymph nodes, *CRM* circumferential resection margin, *pCR* pathological complete response

pStage 4 includes patients with distant metastasis detected during chemoradiotherapy. The crude pCR rates were 20.3% in the TEGAFIRI group and 9.1% in the UFT group. The standardized pCR rates through IPW were 24.3% (95% CI: 12.9 – 35.7%) in the TEGAFIRI group and 8.8% (95% CI: 5.4–12.3%) in the UFT group, with a difference in pCR of 15.4% (95% CI: 4.3–27.8%), *P* = 0.010.

### Survival

**Fig. 2:**
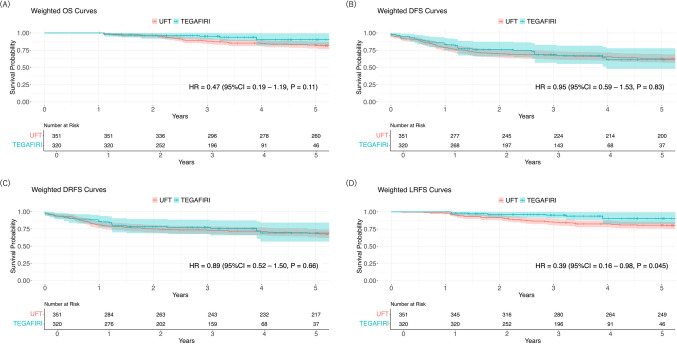
Confounder-adjusted Kaplan–Meier curves for the TEGAFIRI group and UFT group. **A** Overall survival. **B** Disease-free survival. **C** Distant recurrence-free survival. **D** Local recurrence-free survival

Survival analysis was performed on the date of surgery. Patients with distant metastases before surgery (disease progression during CRT) were considered to have recurrence at the time of surgery. The median follow-up period was 3.2 and 7.0 years for the TEGAFIRI and UFT groups. The confounder-adjusted Kaplan–Meier curves for OS, DFS, DRFS, and LRFS are shown in Fig. 2A–D. The weighted 3-year OS, DFS, and DRFS rates showed no significant differences between the groups, with 88.1% vs. 95.2%, 67.2% vs. 68.5%, and 73.1% vs. 77.2% for UFT and TEGAFIRI, respectively. However, the weighted 3-year LRFS rate was significantly higher in the TEGAFIRI group, at 95.2% compared to 84.9% in the UFT group. The results of the Cox regression analysis for risk factors associated with OS are shown in Table 5. In addition, none of the factors listed in the table were significant for DFS. However, clinical T4 (HR = 1.77, 95% CI: 1.06 – 2.98, *P* = 0.03) and clinical LLN positivity (HR = 1.59, 95% CI: 1.03 – 2.45, *P* = 0.04) were significant risk factors for DRFS, while pCR (HR = 0.45, 95% CI: 0.21 – 0.98, *P* = 0.045) was a protective factor. For LRFS, clinical T4 (HR = 2.94, 95% CI: 1.02 – 4.78, *P* < 0.001) was identified as a significant risk factor.

**Table 5:** Risk factors for OS by Cox regression model

Clinicopathological factor	HR (95% CI)	*P*
Age	1.00 (0.99–1.04)	0.42
Sex, female	1.07 (0.64–1.78)	0.81
Clinical T4	2.07 (1.05–4.12)	0.04
Clinical MesoLN positive	1.23 (0.73–2.06)	0.44
Clinical LLN positive	1.83 (1.04–3.23)	0.04
Regimen, TEGAFIRI	0.50 (0.20–1.24)	0.13
CRM positive or close	3.81 (1.22–11.9)	0.02
pCR	0.56 (0.20–1.57)	0.27

*OS* overall survival rate, *HR* hazard ratio, *CI* confidence interval, *MesoLN* mesorectal lymph nodes, *LLN* lateral lymph nodes, *CRM* circumferential resection margin, *pCR* pathological complete response

## Discussion

This study reports the outcomes of patients who underwent preoperative CRT with the TEGAFIRI regimen, followed by radical surgery. A retrospective comparison with conventional treatments suggested that TEGAFIRI might be a superior option, as indicated by the increased pCR rate, which contributes significantly to the reduction in LR.

Improving pCR rates has long been an important goal [17, 18]. Clinical trials have investigated the addition of oxaliplatin and irinotecan to oral fluoropyrimidines to enhance radiosensitivity and control distant metastases. However, except for the CAO/ARO/AIO-04 trial [2, 19], oxaliplatin did not improve the pCR rates [20–23]. The interim report of the ARISTOTLE trial showed no significant difference in pCR rates between capecitabine alone and capecitabine with irinotecan (17% vs. 20%, *P* = 0.45); however, grade 3/4 adverse events were significantly higher with irinotecan (12% vs. 21%, *P* = 0.0004) [9].

The focus has recently shifted to controlling potential microdistant metastases for improved prognosis, leading to increased interest in TNT [3–6]; however, TNT poses a challenge due to increased adverse events. Furthermore, the 5-year follow-up results of the RAPIDO trial demonstrated that LR was more frequent in the TNT group, which underwent consolidation after short-course CRT, compared to the CRT group (44/431 [10%] vs. 26/428 [6%]; *P* = 0.027). Additionally, breached mesorectum was observed more often in the TNT group (9/44 [21%] vs. 1/26 [4%]; *P* = 0.048). TNT has been identified as a significant predictor of LR, potentially due to prolonged intervals between RT and surgery, which increases surgical difficulty [7].

Before the advent of TNT, we conducted a phase I/II trial of TEGAFIRI-based CRT and reported its safety and high pCR rate (22.7%) [11]. Consequently, we adopted TEGAFIRI as the first-line treatment, accumulating 81 consecutive patients with a pCR rate of 20% and no LR during the observation period. Moreover, a significant proportion of patients achieved grade 2 or 3 responses compared with conventional treatment.

The incidence of adverse events differed between the UFT and TEGAFIRI groups, with the TEGAFIRI group generally showing higher rates of all-grade adverse events. Significant differences were observed for most adverse events, with notably higher incidence rates in the TEGAFIRI group. Grade 3 or higher adverse events were infrequent in both groups, although the incidence of certain severe events, such as leukopenia, was higher in the TEGAFIRI group. Despite this, the overall frequency of grade 3 or higher events remained within a clinically acceptable range. However, the impact of these adverse events on patients’ quality of life (QOL) was not assessed in this study, and future studies should evaluate it.

TEGAFIRI was associated with grade 3 or higher adverse events in 15.2% of patients (13.6% in the crude data), which is lower than the rates reported in TNT studies. The main severe adverse events included leukopenia, diarrhea, and pain in the perineal region, but dose reductions or treatment interruptions were relatively uncommon. Biweekly administration of irinotecan and UFT is a key feature of this regimen. Hospitalization for irinotecan administration allowed for thorough monitoring and management of side effects by a multidisciplinary team.

Irinotecan is an important chemotherapeutic agent for colorectal cancer. The dose used as the radiosensitizer differs from that used for recurrent colorectal cancer. For instance, irinotecan doses in recurrent colorectal cancer regimens, such as FOLFIRI and FOLFOXIRI, are 150 and 165 mg/m^2^, respectively. In the PRODIGE 23 trial, which used FOLFIRINOX as part of TNT, the irinotecan dose was 180 mg/m^2^ [3]. In phase II trials combining irinotecan with CRT, doses ranged from 200 to 240 mg/m^2^ [24–27]; however, our regimen used a total dose of 320 mg/m^2^, effectively balancing the efficacy and adverse events. The biweekly schedule may have matched the long-course RT regimen, maximizing the radiosensitizing effect.

The OS and DFS with TEGAFIRI were comparable to those with the conventional UFT regimen. Notably, the LR (crude) was 0% with a median follow-up over 3 years, which is likely attributable to the achievement of clear circumferential resection margins through precise TME. Recently, there has been a tendency to focus more on distant recurrence than pCR rates or LR to improve prognosis. The original concept of TNT was to introduce adjuvant chemotherapy, which is often neglected, to suppress distant recurrence. However, there are concerns that extending the period until surgery increases its difficulty. Therefore, appropriate case selection is crucial for avoiding overtreatment and the associated adverse effects [28]. CRT combined with the TEGAFIRI regimen and selective lateral lymph node dissection could be an important treatment option for LARC.

This study had some limitations. First, this was a retrospective study. Although TEGAFIRI was the first-choice treatment for all patients after study initiation, minimizing selection bias, the conventional cohort was selected from pre-TEGAFIRI patients, potentially introducing a temporal bias. This bias is particularly relevant when interpreting overall survival, as patients in the TEGAFIRI group were treated in a later period, during which advances in diagnostic and treatment modalities may have naturally contributed to improved survival outcomes [29]. Second, the small sample size and differences in the CRT to surgery intervals and disease stage between the groups could have influenced the histological effects and outcomes. Advances in diagnostic modalities may have improved preoperative staging accuracy in the TEGAFIRI group. To minimize the impact of these limitations, we adjusted for background factors using the PS. Given the limited number of pCR cases, we believe that reducing the total number of cases by matching would be undesirable; therefore, we employed IPW with the TEGAFIRI group as a standardized population. Using data from all cases while adjusting for background factors, we demonstrated that the pCR rate was significantly higher in the TEGAFIRI group. Third, this study could not report cT3 subgroups, and information on extramural venous invasion (EMVI) and clinical CRM status was unavailable or inconsistently assessed. Additionally, the quality of TME was not evaluated using the Mercury criteria, which may have influenced local control outcomes. Fourth, there was a difference in the use of adjuvant chemotherapy between the two groups. Since the presence or absence of adjuvant chemotherapy did not affect the primary endpoint, pCR, it was not included as a covariate in the IPW adjustment. Similarly, for the prognostic analysis, adjuvant treatments were heterogeneous due to historical background and, therefore, were not included as adjustment factors in the analysis. We decided to conclude the study in September 2023 because we had initiated a TNT study [30], which combined TEGAFIRI with consolidation CAPOX/FOLFOX for most cT4 or cLLND positive cases. These cases, as indicated by our results, suggest that this group has a high risk of distant recurrence. This decision was based on confidence in the safety of the TEGAFIRI regimen. Therefore, we defined the study period to ensure that subsequent TEGAFIRI cases, which might include less advanced cases not included in the TNT trial, would not bias the study outcomes.

CRT with the TEGAFIRI regimen for LARC demonstrated a high response rate, with a pCR rate of over 20% and with significantly low incidence of LR. Additionally, the regimen effectively minimized adverse events, such as leukopenia and diarrhea. TEGAFIRI shows excellent promise as a treatment regimen for reducing LR and enhancing the curative potential of surgery.

## Data Availability

No datasets were generated or analysed during the current study.
